# Supplementary material for: Dialkyl Carbamoyl Chloride–Coated Dressing Prevents Macrophage and Fibroblast Stimulation via Control of Bacterial Growth: An In Vitro Assay
Source: Microorganisms. 2022 Sep 13;10(9):1825. doi: 10.3390/microorganisms10091825 (PMC9502631; doi:10.3390/microorganisms10091825)
Supplement: Supplementary file 1 [file microorganisms-10-01825-s001.zip › microorganisms-1809877-supplementary.pdf]

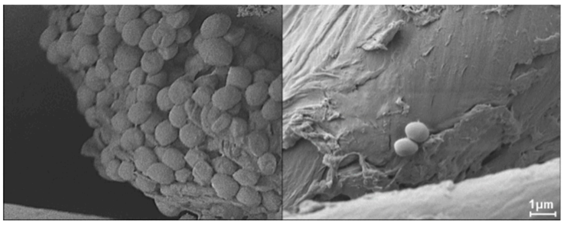

Figure S1. SEM high-magnification photomicrographs. Photomicrographs acquired by SEM of the DACC-coated dressing (left panel) and gauze (right panel) cultured with *S. aureus* in supplemented DMEM. Bacterial cells with the typical size and cocci morphology according to *S. aureus*. In the left panel, cocci are on and embedded in their extracellular matrix.

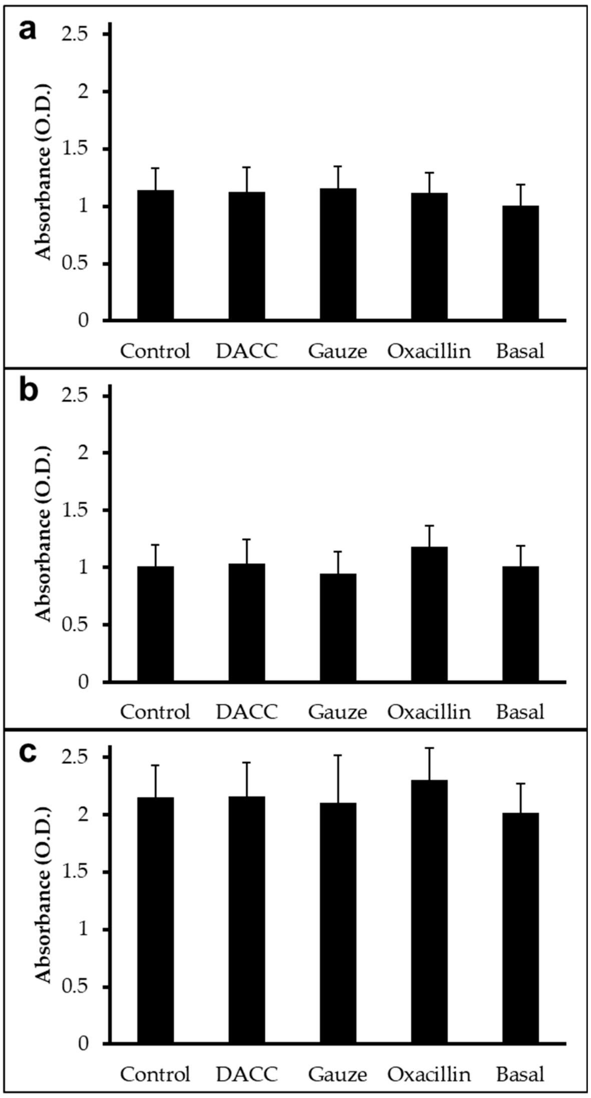

Figure S2. Cell viability assay in fibroblast and macrophage cultures. MTT reduction was evaluated in cultures of fibroblasts (**a**), macrophages (**b**), and co-cultures of fibroblasts and macrophages (**c**) after treatment with the filtered supernatants of *S. aureus* cultured in supplemented DMEM and treated with DACC and gauze dressings. Values are presented as means ± standard deviations. The basal term refers to the conditioned medium from non-unstimulated eukaryotic cultures.
